# Residency Time as an Indicator of Reproductive Restraint in Male Burying Beetles

**DOI:** 10.1371/journal.pone.0109165

**Published:** 2014-10-08

**Authors:** Ashlee N. Smith, Mark C. Belk, J. Curtis Creighton

**Affiliations:** 1 Department of Biological Sciences, Purdue University Calumet, Hammond, Indiana, United States of America; 2 Department of Biology, Brigham Young University, Provo, Utah, United States of America; University of Melbourne, Australia

## Abstract

The cost of reproduction theory posits that there are trade-offs between current and future reproduction because resources that are allocated to current offspring cannot be used for future reproductive opportunities. Two adaptive reproductive strategies have been hypothesized to offset the costs of reproduction and maximize lifetime fitness. The terminal investment hypothesis predicts that as individuals age they will allocate more resources to current reproduction as a response to decreasing residual reproductive value. The reproductive restraint hypotheses predicts that as individuals age they will allocate fewer resources to current reproduction to increase the chance of surviving for an additional reproductive opportunity. In this study, we test for adaptive responses to advancing age in male burying beetles, *Nicrophorus orbicollis*. Burying beetles use facultative biparental care, but the male typically abandons the brood before the female. Previous work in male burying beetles has suggested several factors to explain variation in male residency time, but no study has observed male behavior throughout their entire reproductive lifetimes to determine whether males change residency time in an adaptive way with age. We compared residency time of males that reproduced biparentally, uniparentally, and on different-sized carcasses to determine if they used an adaptive reproductive strategy. Males did not increase residency time as they aged when reproducing biparentally, but decreased residency time with age when reproducing uniparentally. A decrease in parental care with age is consistent with a reproductive restraint strategy. When female age increased over time, males did not increase their residency time to compensate for deteriorating female condition. To our knowledge, this is the first test of adaptive reproductive allocation strategies in male burying beetles.

## Introduction

A fundamental concept of life-history theory is the trade-off between current reproduction and future survival and reproduction [1]. Iteroparous organisms are expected to reserve resources for future reproduction as long as additional reproductive opportunities are possible [2]–[4]. This may act as a constraint on current reproduction because individuals should restrict their current effort to maximize lifetime reproductive success [1]. Individuals are expected to adaptively change the amount of effort that they put into reproduction as they age to maximize their lifetime fitness.

Two adaptive reproductive strategies have been hypothesized. The first strategy, the terminal investment hypothesis, predicts that as individuals age they will increase the amount of resources that they allocate to current reproduction in response to a decrease in residual reproductive value (i.e., future reproductive opportunities) [5]–[6]. The second strategy, the reproductive restraint hypothesis, predicts that as individuals age they will decrease the amount of resources that they allocate to current reproduction in response to deteriorating physical condition to increase the probability of realizing additional reproductive opportunities, thereby increasing lifetime reproductive success [7]. Both strategies predict changes in allocation to current reproduction based on variation in age. Tests of adaptive allocation of resources have been done almost exclusively on females because typically males only invest in reproduction through the production of gametes. However in biparental species these alternative hypotheses can be evaluated with males as well.

In biparental systems, the costs associated with parental care create a conflict between parents [8]–[9]. Thus, investment in the current reproductive bout by one of the parents may depend on the state of that parent and on the state of the other parent as well. Biparental care systems allow us to test the two hypotheses of adaptive reproductive allocation outlined above, and also to assess whether reproductive investment is determined by the state of the target parent, the other parent, or both.

Burying beetles (*Nicrophorus* sp.) provide an ideal model system for evaluating patterns of reproductive investment in a biparental system. Burying beetles exhibit facultative biparental care but the female parent tends to care for offspring for a longer period of time than the male parent [10]–[12]. How long parents stay with the offspring and provide parental care (i.e., residency time) is a measure of the cost expended on the current reproductive bout. Several studies have proposed causes for variation in male residency time (e.g., carcass size [10]–[11], [13]–[14]; carcass depletion [15]–[17]; female absence [11]; male body size [18]–[19]; competition [18], [20]; and residual reproductive value [21]). However, male residency time may be affected by multiple causal factors, including the state of both the male and the female.

In burying beetles, residency time can be used to determine male reproductive investment strategy. A terminal investment strategy should show an increase in male residency time with age because this would indicate that males are increasing their investment in current reproduction as their residual reproductive value decreases. In contrast, a reproductive restraint strategy should show a decrease in residency time with age because this would indicate that males are decreasing their investment in current reproduction to save energy for additional reproductive opportunities. If males are not using an adaptive strategy to allocate resources to reproduction with age, we would expect male residency time to remain constant.

To identify determinants of residency time in male burying beetles, and to test for the pattern of reproductive allocation with age, we manipulated characteristics of males and females, the resource on which they reproduce, and the parental care strategy (biparental versus uniparental) and then measured resulting residency times. This study is unique because we allowed individuals to reproduce throughout their lifetimes to show changes in allocation of resources that occur with age in response to experimental factors. This design provided us an opportunity to test for determinants of male residency time, and simultaneously to test for differences in reproductive strategy among males (i.e., terminal investment and reproductive restraint). We address two specific questions: (1) Under what conditions is the pattern of male residency time consistent with the terminal investment hypothesis (i.e., increasing with age), the reproductive restraint hypothesis (i.e., decreasing with age), or neither (i.e., no age related change). (2) How does male body size, male age, female body size, female age, size of reproductive resource, and parental care type, or their interactions influence male residency time?

## Methods

### Natural History of *Nicrophorus orbicollis*

*Nicrophorus orbicollis* parents use small vertebrate carcasses as a food resource for larvae [18]. Both parents prepare the carcass, which involves removal of hair or feathers, formation of the carcass into a ball, application of anal and oral secretions that prevent decay, and burial of the prepared carcass. Although *N. orbicollis* typically raises offspring biparentally, both sexes are capable of performing all parental duties in the absence of a partner [22]. The female lays eggs in the soil around the buried carcass, which typically hatch approximately 5–7 days after carcass preparation begins. After hatching, the larvae move to a small hole in the carcass made by the parents. Larvae feed directly from the carcass as well as by receiving regurgitated partially digested carrion from both parents [23]–[24]. The male typically abandons the brood 2–5 days after the larvae hatch, but the female remains with the brood for approximately 7 days, after which the larvae disperse into the soil to pupate [11], [18]. Both parents regulate brood size through filial cannibalism [13], [25] resulting in a positive correlation between offspring number and carcass size [13], [26]–[27].

### Source of Burying Beetles

Burying beetles used in our experiments were captured in central Wisconsin during summers from 2009 to 2011 using pitfall traps baited with aged chicken. Wild-caught pairs were placed on 30-g mouse carcasses and allowed to breed to generate the laboratory population. All mice used in experiments were purchased frozen from a commercial vendor. The date of eclosion was recorded for all laboratory-bred beetles, and all beetles used in these experiments were F_1_ or F_2_ offspring of wild-caught beetles. They were placed individually in small plastic containers (15.6×11.6×6.7 cm) with *ad libitum* raw chicken liver and maintained on a 14∶10 h light:dark cycle. These experiments were run concurrently at Purdue University Calumet from 2009 to 2012.

### Experimental Design

#### Test for Effects of Male Traits

The purpose of this experiment was to evaluate the effect of parental type, male age, and resource quality on the amount of time that males remained with a brood. Parental type was either biparental or uniparental. Resource quality was either low (using 20-g carcasses) or high (using 30-g carcasses). Twelve replicates were completed for each of the two parental types using each of the two resource treatments. Each replicate consisted of all reproductive bouts for a given male during the entire lifetime.

**Figure 1:**
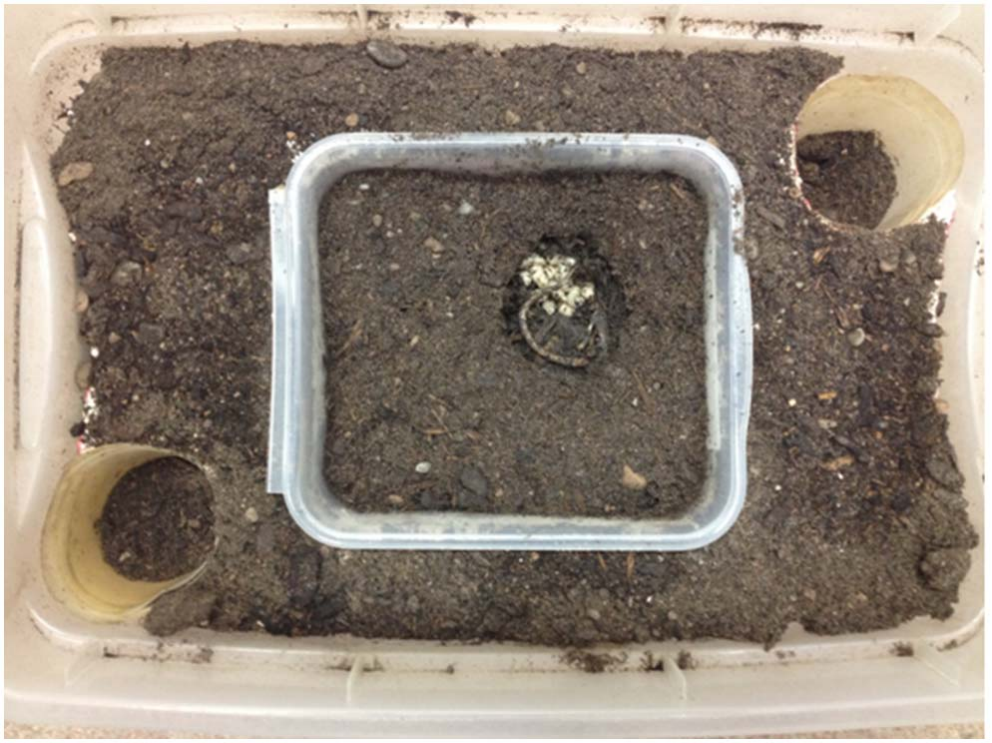
An abandonment chamber used in the experiment. The brood container is the small white container in the center and the abandonment cups are in diagonal corners. A carcass with third instar larvae can also be seen in the container.

We randomly paired a genetically unrelated virgin male and female aged 28–35 days old to begin each trial. The mass, pronotum width, and date of eclosion were recorded for each individual. Male masses were measured using an analytical balance up to 4 decimal places. Male pronotum widths were measured using digital calipers up to 2 decimal places and varied from about 5-mm to 8-mm to encompass the range of body sizes that is seen under natural conditions (Smith, personal observation). The pair was placed in a small brood container (16.5×15×9-cm) filled with 6-cm of moist soil and either a 20-g (±1.0-g) or 30-g (±1.0-g) mouse carcass. The containers were kept in an environmental chamber at 21°C on a 14∶10 h light:dark cycle. To assess residence time and allow free movement of the parents we used the following experimental setup. The broods were checked daily, and after larvae arrived on the carcass the lid of the small brood container was removed and the container was placed in an abandonment chamber (37.5×25.5×14.5-cm) flush with an elevated, Styrofoam platform. Two cups (diameter: 8-cm, height: 9.5-cm) were placed in diagonal corners of the larger abandonment chamber, again, flush with the elevated ledge (See Figure 1). A thin layer of moist dirt was spread on the ledge and 2-cm of moist dirt was placed in each of the cups. Four to five moist paper towels were placed on top of the dirt in the small brood container to maintain moisture. If the replicate was a uniparental trial, the female was removed from the brood container at the time of larvae arrival to the carcass, but if the replicate was a biparental trial, both parents were allowed to remain in the brood container. The number of larvae was counted each day, and the cups in each corner of the container were checked to see if an adult had abandoned the brood. If a parent was found in a cup, its mass and the date were recorded. It was then placed back in the small container with the brood. If the parent abandoned the brood for a second time, it was removed from the experiment. The initial date and mass were used to calculate residency time and mass change, respectively. If the abandoning parent was the male he was placed on commercially bought *ad libitum* chicken liver, then set up to breed again two days later with a genetically unrelated female aged 28–35 days old that had not previously mated. If the male parent remained with the brood when the larvae dispersed into the soil, he was placed on commercially bought *ad libitum* chicken liver, and then given a new carcass of the same size two days later with a genetically unrelated female aged 28–35 days old that had not previously mated. The cycle continued for each male until death.

#### Test for Effects of Female Traits

This experiment was set up and initially run in the same way as the previous experiment, including the two carcass size treatments and the placement of the breeding container in the abandonment chamber. However, this experiment only consisted of a biparental treatment where females aged over time to allow us to test for the effects of female age and physical deterioration with additional reproductive attempts on male residency time. Each female beetle was re-mated with an unrelated, virgin male aged 28–35 days old for each reproductive attempt and observed until death as described above. Twelve replicates were completed for each of the two carcass size treatments.

### Statistical Analyses

To determine how male residency time was affected by treatments and male and female characteristics, we used a mixed model analysis of variance in two separate analyses. For both analyses we use the GLIMMIX procedure in SAS version 9.3 (SAS Institute Incorporated, Cary, NC, USA). In both analyses the response variable was number of days the male spent on the carcass. In the first analysis we were interested in determining how male characteristics, carcass size, and parental type affected residency time. Our predictor variables were parental type (uniparental vs. biparental), carcass quality (20 g or 30 g carcass), and the covariates male age, and male body size (pronotum width). We included initial brood size and male mass change on the carcass as covariates in preliminary analyses, but removed them for the final analysis because neither showed significant effects. We also included the interaction between male age and parental type. No other interactions were significant in preliminary analyses, so they were not included in the final analysis. Because this is a repeated measures design, i.e., male residency time was measured for each reproductive bout over its entire lifetime, male ID was specified as a random effect.

In the second analysis we were interested in determining how female characteristics and carcass size affected male residency time. Of course, this analysis only included a biparental care treatment. The predictor variables were carcass size and the covariates female age and female body size. We included the interaction of female age with carcass size. All other interactions were not significant in preliminary analyses, so they were not included in the final analysis. Because this was a repeated measures design, i.e., male residency time was measured for each reproductive bout over each female’s entire lifetime, female ID was specified as a random effect.

## Results

**Figure 2:**
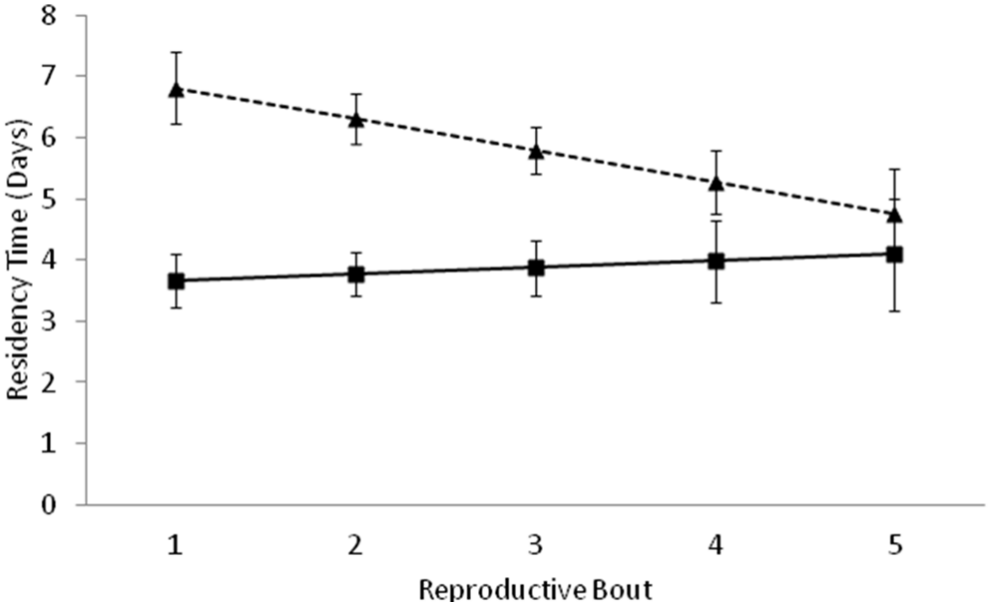
Least-squares means (95% confidence interval) for male residency time per reproductive attempt in the biparental (solid line) and uniparental (dashed line) treatments.

**Figure 3:**
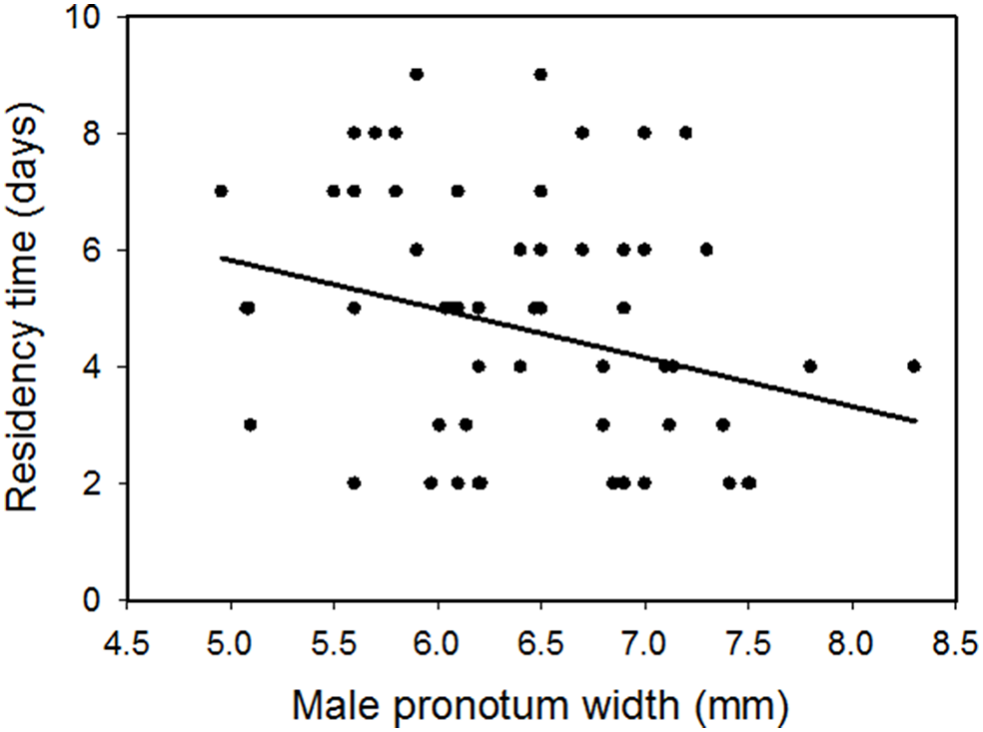
Male residency time plotted on male size for first reproductive bouts of all males. Regression line from analysis of covariance, slope = −0.717, SE = 0.184.

The first set of analyses addressed how male residency time changed with male characteristics. There is a significant difference in male residency time with male age, parental type, and male body size, and the interaction between male age and parental type was significant (Table 1). Initially, uniparental males stayed with the carcass over three days longer than biparental males. Uniparental males decreased their residency time as they aged, but males that raised offspring biparentally did not change residency time as they aged (comparison of confidence intervals, Figure 2) such that by brood five residency time differed by less than one day. Smaller males remained with their broods significantly longer than did larger males (slope of male size on residency time = −0.717, SE = 0.18; Figure 3). Residency time varied by about two days between the smallest and largest males. Carcass size had no effect on male residency time (Table 1).

**Table 1:** Analysis of variance table (ANOVA) for male residency time as a function of male characteristics, carcass size, and parental care type.

Effect	Num df/Den df	F-Value	p-value
Male Age	1/44	4.33	**0.0434**
Carcass Size	1/81	1.36	0.2472
Parental Type	1/81	51.47	**<.0001**
Male Pronotum Width	1/81	15.16	**0.0002**
Male Age*Parental Type	1/81	10.10	**0.0021**

The second set of analyses addressed how male residency time changed with female characteristics. There was no significant effect of female age, female body size, carcass size, or the interaction between female age and carcass size on male residency time (Table 2).

**Table 2:** Analysis of variance table (ANOVA) for male residency time as a function of female characteristics and carcass size.

Effect	Num df/Den df	F-Value	p-value
Female Age	1/21	2.62	0.1207
Carcass Size	1/45	0.00	0.9512
Female Pronotum Width	1/45	0.19	0.6647
Female Age*Carcass Size	1/45	0.02	0.8987

## Discussion

This experiment allowed us a unique opportunity to use a well-documented male behavior as an indicator of adaptive life history decisions. We found no evidence for a terminal investment strategy in males; instead, the behavior of uniparental males across their lifetimes is consistent with a reproductive restraint strategy for investing in reproduction. Male residency time decreased with age when raising offspring uniparentally from 7 days in their first reproductive attempt to 5 days by their fifth reproductive attempt (Figure 2). Larvae took about 7 days to complete development, so younger uniparental males remained with offspring throughout their entire development time. Larvae take 2–3 days to reach the third instar, and are largely independent of regurgitations from the parents by day 5 (Creighton, personal observation). Older males that abandoned their broods after 5 days could be confident that their offspring would survive. The behavior of males under uniparental reproductive conditions is consistent with a reproductive restraint strategy in that males reduced their level of effort in response to their own advancing age and physical state. Young males might have remained with their broods for the entire duration of parental care to compensate for their loss of their partners. However as males aged, they decreased their level of effort to the minimum required to ensure larvae survival when raising offspring alone. By leaving earlier, older males increase their chances of reproducing again, which supports the reproductive restraint hypothesis as proposed by McNamara et al. [7]. This experimental design does not allow us to differentiate between reproductive restraint and senescence effects, but the pattern of residency time is clearly not consistent with a terminal investment strategy.

When male age increased over time under biparental conditions, male residency remained low regardless of male age (Figure 2). In this scenario males invest the minimum amount of effort into reproduction that is required to ensure that the brood is successful. However, the effects of the presence of a female partner and male reproductive strategy cannot be separated in this experiment. Males appear to behave conservatively throughout their lives when reproducing biparentally. This may be due to a lack of need for assistance by the female, which would indicate that an adaptive reproductive strategy is not used by males in this situation. However, this may also be to save energy for additional reproductive opportunities, which would indicate reproductive restraint, but the presence of a female confounds the result as it clearly changes male behavior.

Under biparental and uniparental conditions male residency time decreased with increasing male size. At the extremes, the smallest males remained with their broods an average of 2 days longer than did largest males (Figure 3). This observation is consistent with the idea that larger males abandon their partners earlier because their chances of securing another carcass are higher [19], a behavior that has also been demonstrated in pine engraver beetles [28]–[29]. In burying beetles, there is intense intrasexual competition for carcasses, with the largest individual typically winning dominance on the carcass [30]–[35]. Therefore, smaller males may gain a fitness advantage by remaining with a brood longer as opposed to leaving and searching for a new carcass because their chances of winning another carcass are low.

Males typically abandon their offspring, while females remain with the brood until larvae disperse into the soil to continue development. In our study, male *N. orbicollis* remain with a brood longer when they are left to raise offspring alone than when a female is present. A similar result was found in field studies done by Trumbo [11], as well as laboratory experiments [36]–[37]. It has been suggested that males remain with a brood until the carcass is no longer valuable to conspecifics due to depletion of the resource [16]–[17], about 4–5 days after larvae hatch, therefore eliminating the chance of a takeover [15]. Our results support this idea because males that cared for offspring with a female remained with offspring 3–4 days after larvae hatched. However when the female was removed from the brood, the male did not abandon. Without the female present, the male must provide extended parental care that is typically provided by the female.

Males did not change their residency time in response to their partner’s age when they raised offspring biparentally. As reproducing female burying beetles near the end of their lives, their quality of care declines as measured by speed and efficiency (Smith, personal observation). This reduction in care is similar to what one sees as a result of experimental handicapping, where weights are added to the elytra of beetles to reduce their ability to maintain the carcass or care for offspring. Handicapping experiments conducted with *Nicrophorus* has yielded sex and time-dependent results. In a handicapping experiment on *N. orbicollis* during the carcass preparation stage of reproduction, males compensated for reductions in female effort (Creighton et al. *in prep*). In contrast, a handicapping study during the larvae provisioning stage of reproduction on *Nicrophorus quadripuntatus* found that males did not increase their level of effort to compensate for reduced female effort [38]. The handicapping experiments used carcass maintenance and larvae provisioning [38] behaviors to assess changes in investment, while the current study used residency time as an indicator for investment, but parallels between the studies can still be drawn. Male parental care during larvae provisioning may be less important than female care, but not during carcass preparation [18], [39]. Therefore, males would be expected to compensate for the loss of a female in the carcass preparation stage, but the female presence during larvae provisioning would render the male redundant, even if the female was not working at her maximum. Therefore the presence of the female, regardless of her condition, is sufficient for the male to abandon the brood and search for additional reproductive opportunities. In our experiment, the age of only one of the parents varied so that young males could be paired with young or old females and young females could be paired with young or old males. The reproductive strategy of males in the biparental treatment might have changed to terminal investment if both parents were old as an attempt to compensate for the potentially negative effects of having two old parents providing parental care. However, an additional experiment would be necessary to address this question.
